# 2′-Deoxy-5-(hydroxymethyl)cytidine: estimation in human cancer cells with a simple chemosensor†

**DOI:** 10.1039/c8ra08391f

**Published:** 2018-11-29

**Authors:** Himadri Sekhar Sarkar, Shampa Kundu, Sujoy Das, Pulak Kumar Maiti, Sukhendu Mandal, Prithidipa Sahoo

**Affiliations:** Department of Chemistry, Visva-Bharati University Santiniketan-731235 India prithidipa@hotmail.com; Department of Microbiology, University of Calcutta Kolkata-700073 India

## Abstract

A pyrrole-based rhodamine conjugate (CS-1) has been developed and characterized for the selective detection and quantification of 2′-deoxy-5-(hydroxymethyl)cytidine (5hmC) in human cancer cells with a simple chemosensing method.

## Abstract 2

A new chemosensor, CS-1, has been developed and characterized for the selective detection and quantification of 2′-deoxy-5-(hydroxymethyl)cytidine (5hmC) in human cancer cells.

## Graphical abstract

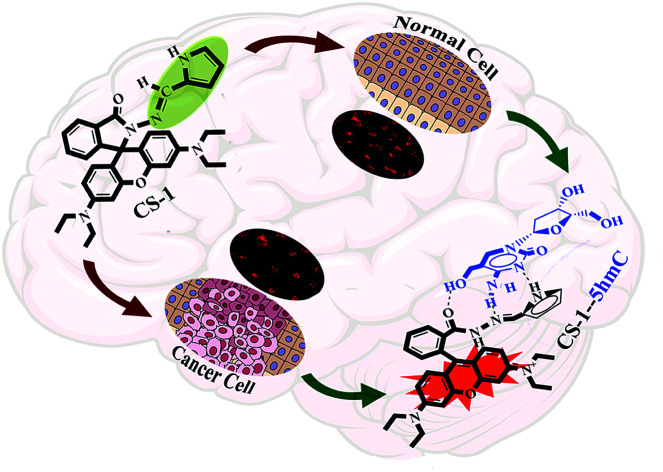

2′-Deoxy-5-(hydroxymethyl)cytidine (5hmC) is found in both neuronal cells and embryonic stem cells. It is a modified pyrimidine and used to quantify DNA hydroxymethylation levels in biological samples^1–3^ as it is capable of producing interstrand cross-links in double-stranded DNA. It is produced through an enzymatic pathway carried out by the Ten-Eleven Translocation (TET1, TET2, TET3) enzymes, iron and 2-oxoglutarate dependent dioxygenase.^4–7^ In the DNA demethylation process, methylcytosine is converted to cytosine and generates 5hmC as an intermediate in the first step of this process which is then further oxidized to 5-formylcytosine (fC) and 5-carboxycytosine (caC) of very low levels compared to the cytosine level.^8^ Though the biological function of 5hmC in the mammalian genome is still not revealed, the presence of a hydroxymethyl group can regulate gene expression (switch ON & OFF). Reports say that in artificial DNA 5hmC is converted to unmodified cytosine when introduced into mammalian cells.^9,10^

Levels of 5hmC substantially vary in different tissues and cells. It is found to be highest in the brain, particularly in nervous system and in moderate percentage in liver, colon, rectum and kidney tissues, whereas it is relatively low in lung and very low in breast and placenta.^11,12^ The percentage of 5hmC content is much less in cancer and tumor tissues compared to the healthy ones. The reason behind this loss is the absence of TET1, TET2, TET3, IDH1, or IDH2 mutations in most of the human cancer cells which means decrease of methylcytosine oxidation.^13–15^ This loss of 5hmC in cancer cells is being used as a diagnostic tool for the detection of early-stage of malignant disease. Few analytical methods^16–19^ such as glucosyltransferase assays, tungsten-based oxidation systems, and TET-assisted bisulfite sequencing (TAB-Seq) or oxidative bisulfite sequencing (oxBS-Seq) protocols are now developed to differentiate 5hmC from other nucleotide which are naturally occurred. There are also few methods such as liquid chromatography/tandem mass spectroscopy (LC/MS-MS), which determine the level of 5hmC in mammalian cancer cell.^20–22^ However, these procedures are highly toxic and expensive due to requirement of catalyzation through enzymes or heavy metal ion and these techniques require expertise, facilities, much time and costs even beyond standard DNA sequencing. As a result, these detection techniques are currently inappropriate for the high-throughput screening of genome-wide 5hmC levels (performance comparison is shown in Table S1, ESI†).

Among all reputed methods fluorescence detection method using chemosensors is significantly important due to its indispensable role in medicinal and biological applications.^23–27^ Chemosensors have been effectively explored to monitor biochemical processes and assays through *in situ* analysis in living systems and abiotic samples with much less time and cost.

In this contribution we prepared and characterize (Scheme S1 and Fig. S1–S3, ESI†) a pyrrole–rhodamine based chemosensor (CS-1) which shows efficient and selective fluorescence signal for 5hmC in aqueous medium (Scheme 1). A transparent single crystal of CS-1 (Fig. 1) was obtained by slow evaporation of the solvent from a solution of CS-1 in CH_3_CN. It crystallizes as monoclinic with space group *P*2_1_/*n* (Fig. S4 and Table S2, ESI†).

**Scheme 1:**
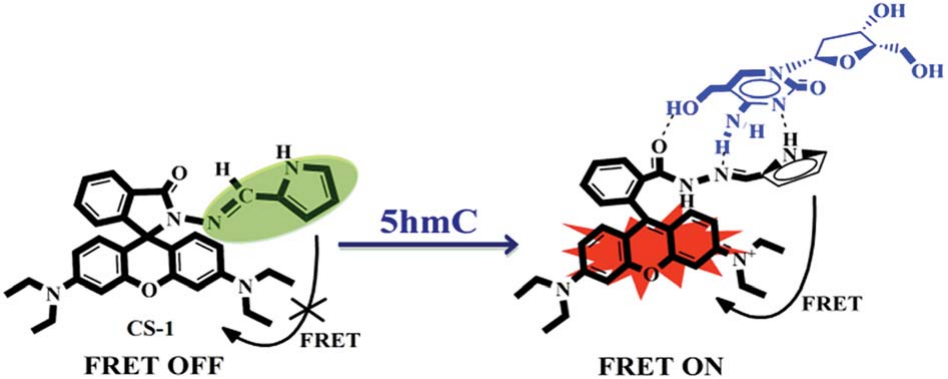
5hmC-induced FRET OFF–ON mechanism of the chemosensor CS-1.

**Fig. 1:**
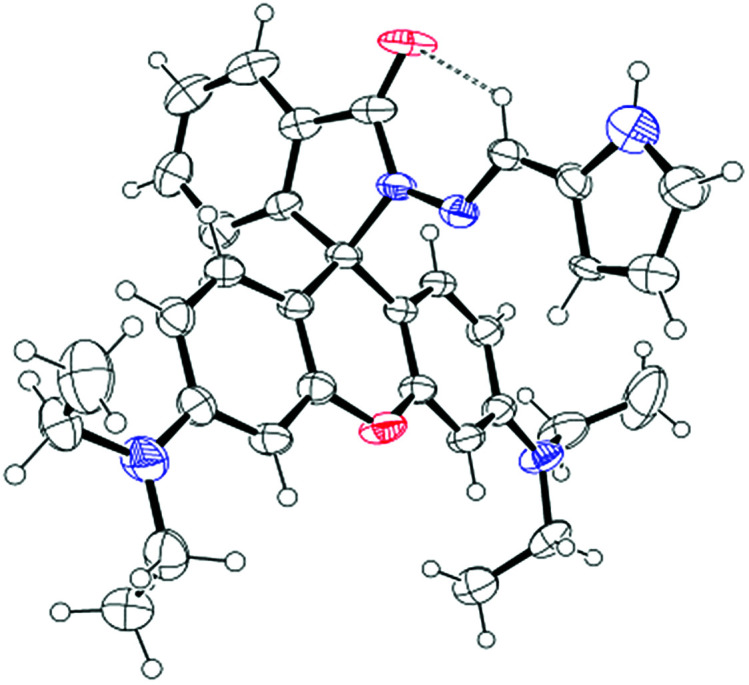
ORTEP diagram of CS-1 (ellipsoids are drawn at 40% probability level).

Spectrophotometric and spectrofluorimetric titrations were carried out to understand the CS-1–5hmC interaction with 1 : 1 binding stoichiometry (Fig. S5, ESI†) upon adding varying concentrations of 5hmC to a fixed concentration of CS-1 (1 μM) in aqueous medium at neutral pH. Upon the addition of increasing concentrations of the 5hmC, a clear absorption band (*K*_a_ = 4.47 × 10^5^ M^−1^, Fig. S6, ESI†) appeared to be centered at 556 nm with increasing intensity (Fig. 2a). On the other hand, for the fluorescence emission spectra of CS-1 (Fig. 2b), upon irradiation at 325 nm, an emission maxima at 390 nm was observed, which was attributed to the fluorescence emission from the donor unit *i.e.* the pyrrole moiety of CS-1. When 5hmC were added, due to rhodamine moiety CS-1 showed a 95-fold increase in fluorescence at 565 nm (*K*_a_ = 4.61 × 10^5^ M^−1^, Fig. S7, ESI†) with the detection limit of 8 nM (Fig. S8, ESI†). The binding of 5hmC induces opening of the spirolactam ring in CS-1, inducing a shift of the emission spectrum. Subsequently, increased overlap between the emission of the energy-donor (pyrrole) and the absorption of the energy-acceptor (rhodamine) greatly enhances the intramolecular FRET process,^28,29^ producing an emission from the energy acceptor unit in CS-1.

**Fig. 2:**
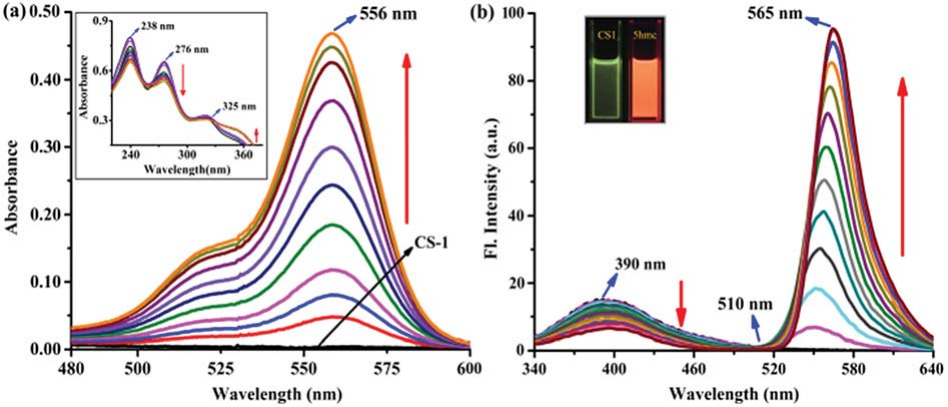
(a) UV-vis absorption spectra of CS-1 (1 μM) upon gradual addition of 5hmC up to 1.2 equiv. in H_2_O–CH_3_CN (15 : 1, v/v) at neutral pH. (b) Fluorescence emission spectra of CS-1 (1 μM) upon addition of 1.2 equiv. of 5hmC in H_2_O–CH_3_CN (15 : 1, v/v) at neutral pH (*λ*_ex_ = 325 nm).

In order to establish the sensing selectivity of the chemosensor CS-1, parallel experimentations were carried out with other pyrimidine/purine derivatives such as 5-methylcytosine, cytosine, cytidine, thymine, uracil, 5-hydroxymethyluracil, adenine and guanine. Comparing with other pyrimidine/purine derivatives the abrupt fluorescence enhancement was found upon addition of 5hmC to CS-1 while others do not make any fluorescence changes under UV lamp (Fig. 3, lower panel). Furthermore, the prominent color change from colorless to deep pink allows 5hmC to be detected by naked eye (Fig. 3, upper panel). The above observation shows consistency with the fluorescence titration experiments where no such binding of CS-1 with other pyrimidine/purine derivatives was found (Fig. S9, ESI†).

**Fig. 3:**
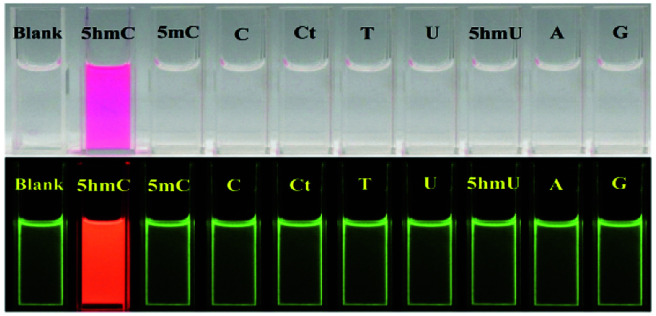
Visible color (top) and fluorescence changes (bottom) of CS-1 (1 μM) in aqueous medium upon addition of 1.2 equiv. of various pyrimidine/purine derivatives (*λ*_ex_ = 325 nm) in H_2_O–CH_3_CN (15 : 1, v/v) at neutral pH.

pH titration reveals that CS-1 becomes fluorescent below pH 5 due to the spirolactam ring opening of rhodamine. However, it is non-fluorescent at pH range of 5–13. Upon addition of 5hmC to CS-1 shows deep red fluorescence in the pH range of 5–8 (Fig. S10, ESI†). Considering the biological application and the practical applicability of the chemosensor pH 7.4 has been preferred to accomplish all experiments successfully.

In ^1^H NMR titration (Fig. S11, ESI†), the most interesting feature is the continuous downfield shift of aromatic protons on the pyrrole moiety of CS-1 upon gradual addition of 5hmC. This may be explained as the decrease in electron density of the pyrrole moiety upon binding with 5hmC through hydrogen bonding. Xanthene protons to be shifted downfield upon spirolactam ring opening indicates the probe to coordinate with 5hmC and electrons are accumulated around 5hmC. In ^13^C NMR titration the spiro cycle carbon peak at 65 ppm was shifted to 138 ppm along with a little downfield shift of the aromatic region of CS-1 (Fig. S12, ESI†). This coordination led to the spiro cycle opening and changes to the absorption and emission spectra, further evident by mass spectrometry (Fig. S13, ESI†), which corroborates the stronger interaction of CS-1 with 5hmC.

The experimental findings were validated by density functional theory (DFT) calculations using the 6-31G+(d,p) method basis set implemented at Gaussian 09 program. Energy optimization calculations presented the conformational changes at the spirolactam position of CS-1 while 5hmC takes part to accommodate a probe molecule. After CS-1–5hmC complexation the energy is minimized by 19.45 kcal from the chemosensor CS-1, indicating a stable complex structure (Fig. 4 and Table S3, ESI†). This theoretical study strongly correlates the experimental findings.

**Fig. 4:**
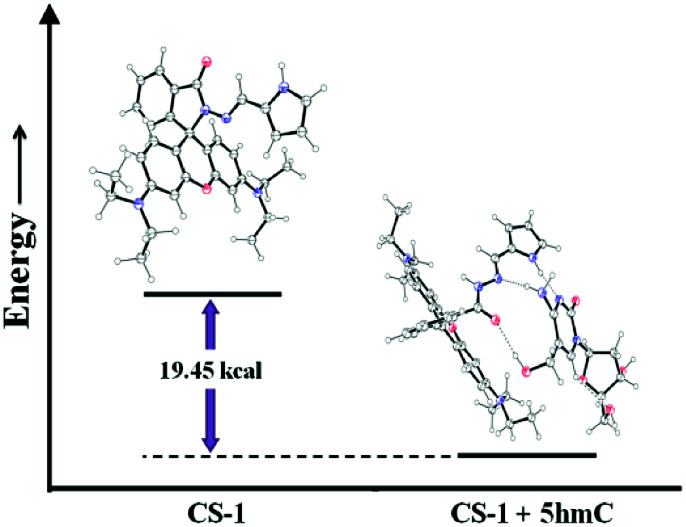
Energy diagram showing the energy differences between CS-1 and CS-1–5hmC complex.

The desirable features of CS-1 such as high sensitivity and high selectivity at physiological pH encouraged us to further evaluate the potential of the chemosensor for imaging 5hmC in live cells (Fig. 5). A549 cells (Human cancer cell A549, ATCC no. CCL-185) treated with CS-1 (1 μM) exhibited weak fluorescence, whereas a deep red fluorescence signal was observed in the cells stained with CS-1 (1 μM) and 5hmC (10 μM), which is in good agreement with the FRET OFF–ON profile of the chemosensor CS-1 in presence of 5hmC, thus corroborating the in-solution observation (Fig. S14, ESI†). Cytotoxicity assay measurement shows that the chemosensor CS-1 does not have any toxicity on the tested cells and CS-1–5hmC complex does not exert any significant adverse effect on cell viability at tested concentrations (Fig. S15, ESI†). As far as we are aware, this is the first report where we are executing the possible use of the pyrrole–rhodamine based chemosensor for selective recognition of 5hmC in living cells. These findings open an avenue for future biomedical applications of the chemosensor to recognize 5hmC.

**Fig. 5:**
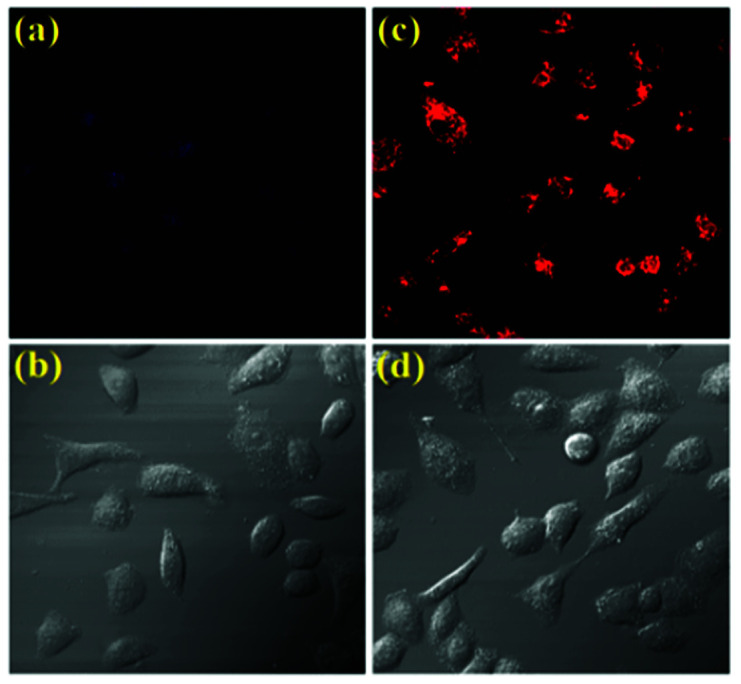
Confocal microscopic images of A549 cells treated with CS-1 and 5hmC. (a) Cells treated with only CS-1 at 1 μM concentration. (b) Bright field image of (a). (c) Cells treated with CS-1 and 5hmC at concentration 10 μM. (d) Bright field image of (c). All images were acquired with a 60× objective lens with the applied wavelengths: For (a) and (b), *E*_ex_ = 341 nm, *E*_em_ = 414 nm, filter used: DIDS; for (c) and (d) *E*_ex_ = 550 nm, *E*_em_ = 571 nm, filter used: Rhod-2.

The concentration of 5hmC was also quantified from A549 human cancer cells. Lysate of 10^7^ A549 cells was added to 1 μM of CS-1 and the fluorescence signal was recorded. Presence of 5hmC in these cancer cells was detected with the help of CS-1–5hmC standard fluorescence curve (Fig. 6) using the selective detection ability of the chemosensor CS-1.

**Fig. 6:**
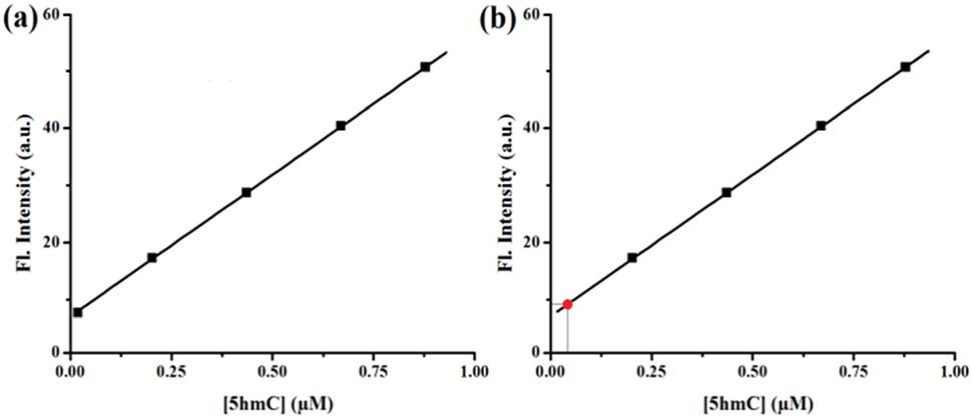
(a) Calibration curve obtained for the estimation of 5hmC. (b) Estimation of the concentration of 5hmC (red point) from the calibration curve.

From the standard curve it was found that the concentration of 5hmC in the tested sample was 0.034 μM present in 16.7 mm^3^ A549 cell volume (Table 1). The above result was authenticated by spiking a real sample with a known concentration of 5hmC and different fluorescence signals were observed by adding known volumes from each sample to the CS-1. The recovery of the spiked samples was estimated to be over 99% (Table S4, ESI†). Assay of 5hmC was further validated from multiple samples of A549 human cancer cells using CS-1. Increasing fold of fluorescence signals was also statistically validated after calculating the *Z*′ value (Table S5, ESI†). All tested samples shows the *Z*′ score value more than 0.9, indicating an optimized and validated assay of 5hmC.

**Table 1:** Quantification of 5hmC in human cancer cell A549

Sample	CS-1 used (μM)	Initial 5hmC used	Addition of exogenous 5hmC (μM)	Amount of 5hmC derived from fluorescence signal (μM)	Fluorescence signal recovery (%)
1	1	5hmC present in 16.7 mm^3^ A549 cell volume	0	0.034	—
2	1		1	1.028	99.4
3	1		3	4.019	99.6
4	1		5	5.012	99.5

## Conclusions

In conclusion, a chemosensor CS-1 has been developed for the rapid detection of 5hmC with low cost. The selective detection and quantification of 5hmC was successfully demonstrated in human cancer cells at neutral pH with very low concentration. With this potentiality of CS-1 one can successfully apply this method to estimate 5hmC in disease cancer tissues and other biological samples of patients with metabolic dysfunction or various carcinomas.

## Conflicts of interest

There is no conflicts to declare.

## Supplementary Material

RA-008-C8RA08391F-s001

RA-008-C8RA08391F-s002
