# Supplementary material for: 2′-Deoxy-5-(hydroxymethyl)cytidine: estimation in human cancer cells with a simple chemosensor
Source: RSC Adv. 2018 Nov 29;8(70):39893–6. doi: 10.1039/c8ra08391f (PMC9091298; doi:10.1039/c8ra08391f)
Supplement: RA-008-C8RA08391F-s001 [file RA-008-C8RA08391F-s001.pdf]

Electronic Supplementary Material (ESI) for RSC Advances.
This journal is © The Royal Society of Chemistry 2018

2′-Deoxy-5-(hydroxymethyl)cytidine: Estimation in human cancer cells by a simple chemosensor

Himadri Sekhar Sarkar,[†] Shampa Kundu,[†] Sujoy Das,[†] Pulak Kumar Maiti,[‡] Sukhendu Mandal[‡] and Prithidipa Sahoo*[†]

[†]Department of Chemistry, Visva-Bharati University, Santiniketan-731235, India.
Email: prithidipa@hotmail.com
[‡]Department of Microbiology, University of Calcutta, Kolkata-700073, India.

Table S1. Performance comparison of existing methods and present method for detection of 5hmC

Method of detection	Detection limit	Response time	Exogenous detection	Endogenous detection	Water soluble	Cost effective	References
Fluorescence based chemosensor	8 nm	Few seconds	Yes	Yes	Yes	Yes	Present Manuscript
LC–MS	ND	ND	Yes	No	No	No	*Angew. Chem. Int. Ed.* **2010**, *49*, 5375.
LC/MS–MS	ND	Few days	Yes	No	No	No	*Cancer Res.* **2011**, *71*, 7360.
β-glucosyl-transferase assays	ND	Several hours	Yes	No	No	No	*Nucleic Acids Research* **2011**, *39*, e55.
Immuno fluorescence method	ND	Several hours	Yes	No	No	No	*Cell* **2012**, *150*, 1135.
Isotope-labelled LC–MS/MS	57 pg/ml	Several minutes	Yes	No	No	No	*Bioanalysis* **2013**, *5*, 839.
Fluorescence method	ND	Several hours	Yes	No	Yes	Yes	*RSC Adv.* **2013**, *3*, 12066.
Spectroscopic method	0.002% of total nucleotides	Several minutes	Yes	No	Yes	No	*Anal. Chem.* **2014**, *86*, 8231.

Experimental Section:

Materials and methods:

Rhodamine B, hydrazine, pyrrole-2-carboxaldehyde and 2'-deoxy-5-(Hydroxymethyl)-cytidine were purchased from Sigma-Aldrich Pvt. Ltd. Unless otherwise mentioned, materials were obtained from commercial suppliers and were used without further purification. Solvents were dried according to standard procedures. Elix Millipore water was used throughout all experiments. ^{1}H and ^{13}C NMR spectra were recorded on a Bruker 400 MHz instrument. For NMR spectra, DMSO-d_6 and for NMR titration DMSO-d_6 and D_2O were used as solvent using

TMS as an internal standard. Chemical shifts are expressed in δ ppm units and $^1H–^1H$ and $^1H–C$ coupling constants in Hz. The mass spectrum (HRMS) was carried out using a micromass Q-TOF Micro™ instrument by using methanol as a solvent. Fluorescence spectra were recorded on a Perkin Elmer Model LS 55 spectrophotometer. UV spectra were recorded on a SHIMADZU UV-3101PC spectrophotometer. Elemental analysis of the compounds was carried out on Perkin-Elmer 2400 series CHNS/O Analyzer. The following abbreviations are used to describe spin multiplicities in ^1H NMR spectra: s = singlet; d = doublet; t = triplet; q= quartet; m = multiplet.

Synthetic Procedure:

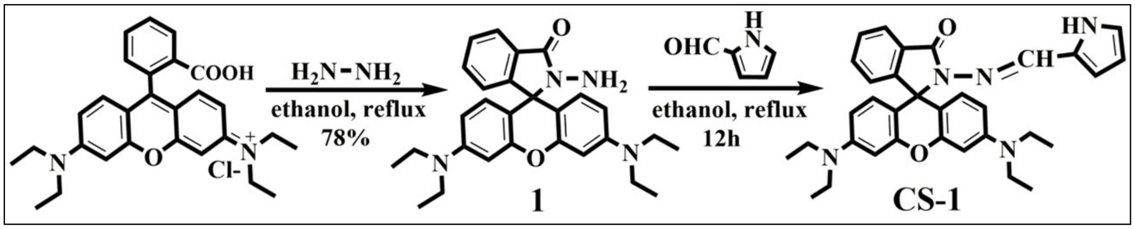

Scheme S1. Synthesis of **CS-1**.

CS-1 was prepared by reacting rhodamine B hydrazide with pyrrole-2-carboxaldehyde (Scheme S1). Rhodamine B hydrazide was synthesized according to literature.[1] Rhodamine B hydrazide (0.46 g, 1 mmol) was dissolved in 20 ml ethanol, then excess dihydroxybenzaldehyde (0.12 g, 1.2 mmol) was added, then the mixture was refluxed for 12 h under N_2 atmosphere. Then, the reaction mixture was poured into water. The solution was extracted with CH_2Cl_2 (3×50 mL), and the combined organic layer was washed with 5% aqueous HCl (50 mL), 10% aqueous Na_2CO_3 (50 mL) and finally with water and then was dried over anhydrous $MgSO_4$. After removing the solvents, the residue was chromatographed on silica gel (220–400 mesh) with chloroform/ethyl acetate = 1:3 v/v as eluent to get the product **CS-1** as pale pink solid with 78% yield. ^1H NMR (400 MHz, DMSO-d_6): d (ppm) = 11.16 (s, 1H), 8.47 (s, 1H), 7.82–7.84 (t, 1H, J = 8 Hz), 7.49–7.52 (m, 2H, J = 12 Hz), 6.98–6.99 (d, 1H, J = 4Hz), 6.811–6.814 (d, 1H, J=1.2 Hz), 6.40–6.44 (m, 4H, J = 16 Hz), 6.31–6.34 (m, 2H, J = 12 Hz), 6.26 (s, 1H), 6.03–6.04 (d, 1H, J = 4Hz), 3.27–3.32 (m, 8H, J = 20 Hz), 1.05–1.08 (t, 12H, J = 12 Hz). ^{13}C NMR (400 MHz, DMSO-d_6): d (ppm) = 163.39, 152.38, 151.95, 148.39, 142.63, 133.33, 128.46, 128.39, 127.47, 127.11, 123.35, 122.78, 122.73, 113.55, 109.38, 108.01, 105.44, 97.51, 65.05, 43.66, 12.44. HRMS (TOF MS): (m/z, %): Calcd. for $C_{33}H_{35}N_5O_2$: 533.66. Found: m/z = 534.6623 (M + H^+; 100%).

NMR spectral studies:

^{1}H NMR of CS-1 in DMSO-d$_6$:

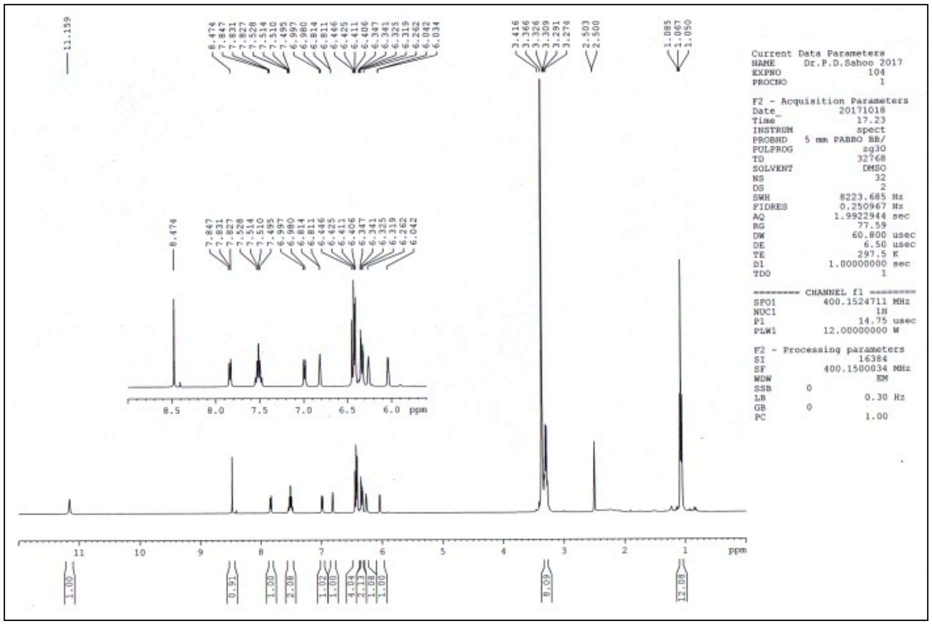

Fig. S1 ^{1}H NMR of **CS-1** in DMSO-d$_6$ (400 MHz).

^{13}C NMR of CS-1 in DMSO-d$_6$:

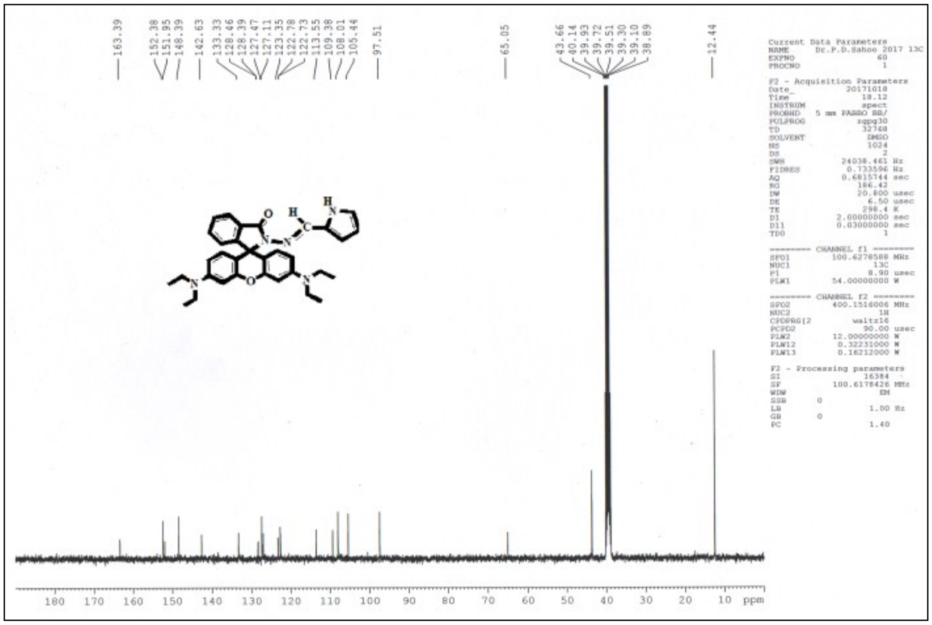

Fig. S2 ^{13}C NMR of **CS-1** in DMSO-d$_6$ (400 MHz).

Mass spectrum of CS-1:

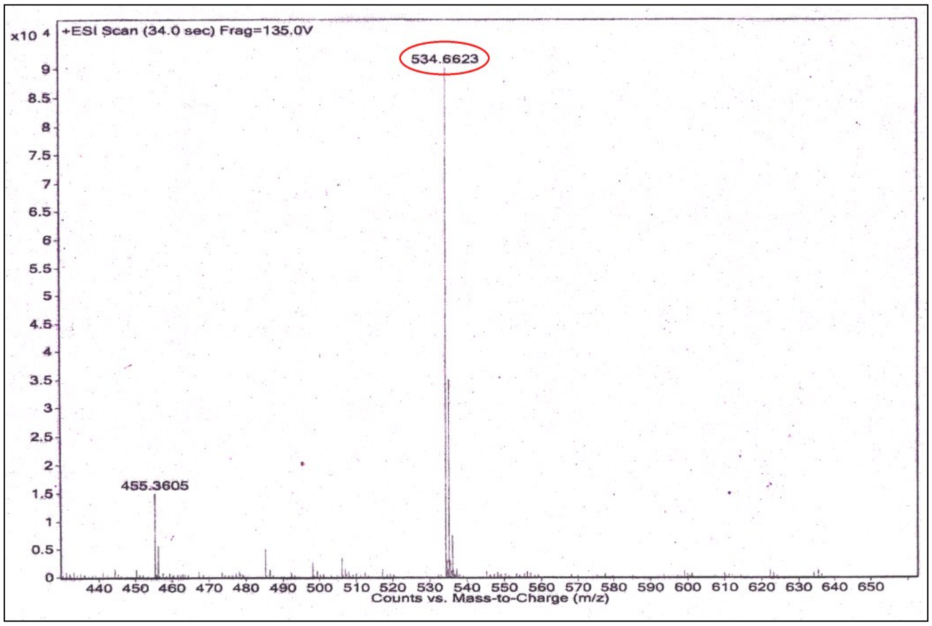

Fig. S3 HRMS of **CS-1**.

X-ray crystal structure of CS-1:

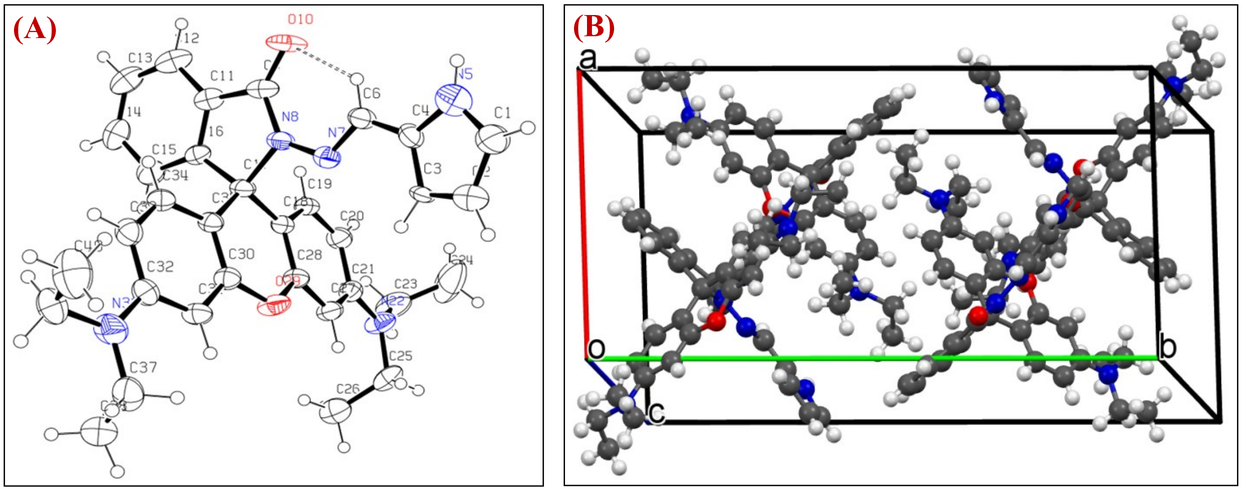

Fig. S4 (A) ORTEP diagram of **CS-1** (with atom numbering). Ellipsoids are drawn at 40% probability level. (B) Crystal packing of **CS-1** along the *b*-axis.

Table S2. Crystallographic data and structural refinement for **CS-1**

Identification code	**CS-1**	
CCDC No.	**1855229**	
Chemical formula	$C_{33}H_{35}N_5O_2$	
Formula weight	533.66 g/mol	
Temperature	296 K	
Wavelength	0.71073 Å	
Crystal system	monoclinic	
Space group	P 21/n	
Unit cell dimensions	a = 11.108(11) Å	α = 90°
	b = 22.604(2) Å	β = 109.349 (5)°
	c = 12.163(12) Å	γ = 90°
Volume	2881.8 (5)$Å^3$	
Z	4	
Density (calculated)	1.230 g/cm^3	
Absorption coefficient	0.078 mm^{-1}	
F(000)	1136.0	
Reflections collected	63481	
Independent reflections	4866 [R(int) = 0.0868]	
Coverage of independent reflections	99.6%	
Refinement method	Full-matrix least-squares on F^2	
Refinement program	SHELXL-2016/4 (Sheldrick, 2016)	
Final R indices	3164 data; $I>2\sigma(I)$	R1 = 0.0720, wR2 = 0.2089
	all data	R1 = 0.1011, wR2 = 0.2456
R.M.S. deviation from mean	0.133 $eÅ^{-3}$	

Preparation of test solutions for UV–vis and fluorescence studies:

A stock solution of **CS-1** (1 μM) was prepared in water–acetonitrile (15:1, v/v). 5hmC solution of concentration 10 μM was prepared in Millipore water. All experiments were carried out in aqueous medium at neutral pH. During titration, each time a 1 μM solution of **CS-1** was filled in a quartz optical cell of 1 cm optical path length and 5hmC stock solution was added into the quartz optical cell gradually by using a micropipette. For all fluorescence measurements, excitations were provided at 325 nm, and emissions were collected from 340 to 640 nm.

Determination of binding stoichiometry by Job's plot:

Judging from the fluorescence titrations, continuous variation method was used to determine the stoichiometric ratios of the host and the specific guest. For **CS-1**–5hmC interaction, the stoichiometry of binding was found to be 1:1 (Figure S5).

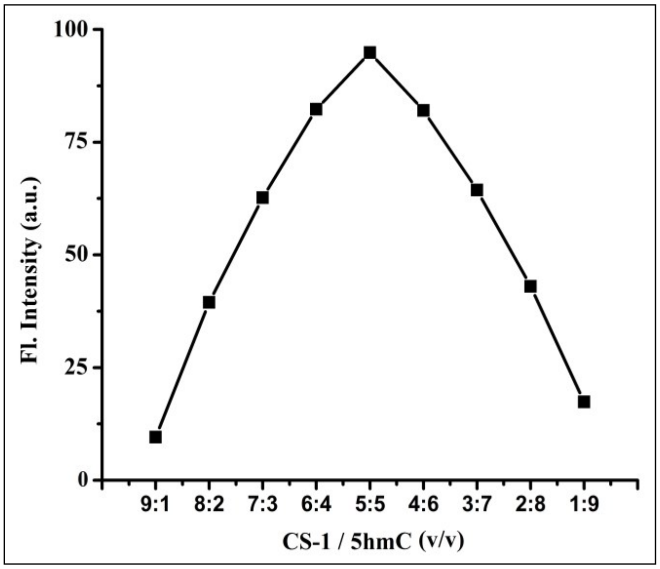

Fig. S5 Job's plot of **CS-1** (1μM) with 5hmC (1μM) in water–acetonitrile (15:1, v/v) at neutral pH by fluorescence method, that indicates 1:1 stoichiometry for **CS-1** with 5hmC.

Evaluation of association constants for the formation of CS-1–5hmC complex:

By UV–vis method:

The substrate binding interaction was calculated according to the Benesi–Hildebrand equation.[2-4]

$$\frac{A_0}{A - A_0} = \left(\frac{\varepsilon_0}{\varepsilon_0 - \varepsilon}\right)^2 \left(\frac{1}{K_B[Substrate]^2} + 1\right) \quad \ldots\ldots\ldots\ldots \text{(i)}$$

Here A_o is the absorbance of receptor in the absence of guest, A is the absorbance recorded in the presence of added guest, ε_0 and ε are the corresponding molar absorption co-efficient and K_B represents the substrate binding interaction with guest.

Binding constant calculation graph (Absorption method):

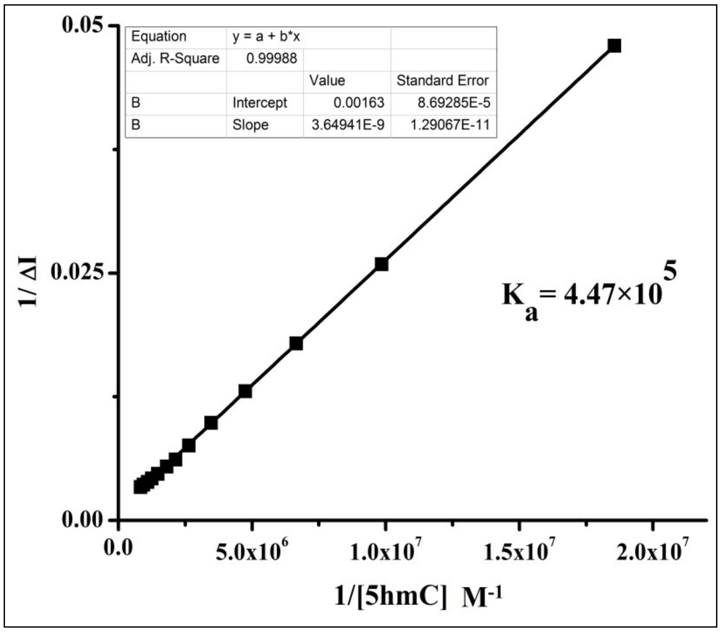

Fig. S6 Linear regression analysis for the calculation of association constant value by UV–vis titration method.

[G] is the concentration of the guest 5hmC and [H] is the concentration of the host **CS-1**.

The association const. (K_a) of **CS-1** for sensing 5hmC was determined from the equation:

$$K_a = \text{intercept/slope}.$$

From the linear fit graph (Figure S6) we get intercept = 0.00163, slope= 3.64941×10^{-9}. Thus we get, $K_a = 0.00163 / (3.64941 \times 10^{-9}) = 4.47 \times 10^5\ M^{-1}$.

By fluorescence method:

Binding constant of the chemosensor **CS-1** was calculated through emission method by using the following equation:

$$1/(I - I_0) = 1/K(I_{max} - I_0)[G] + 1/(I_{max} - I_0) \quad \ldots\ldots\ldots\ldots\ldots \text{(ii)}$$

Where I_0, I_{max}, and I represent the emission intensity of free **CS-1**, the maximum emission intensity observed in presence of added 5hmC at 565 nm (λ_{ex}= 325 nm), and the emission intensity at a certain concentration of 5hmC added, respectively. [G] is the concentration of the guest 5hmC. [H] is the concentration of the host **CS-1**.

Binding constant calculation graph (Fluorescence method):

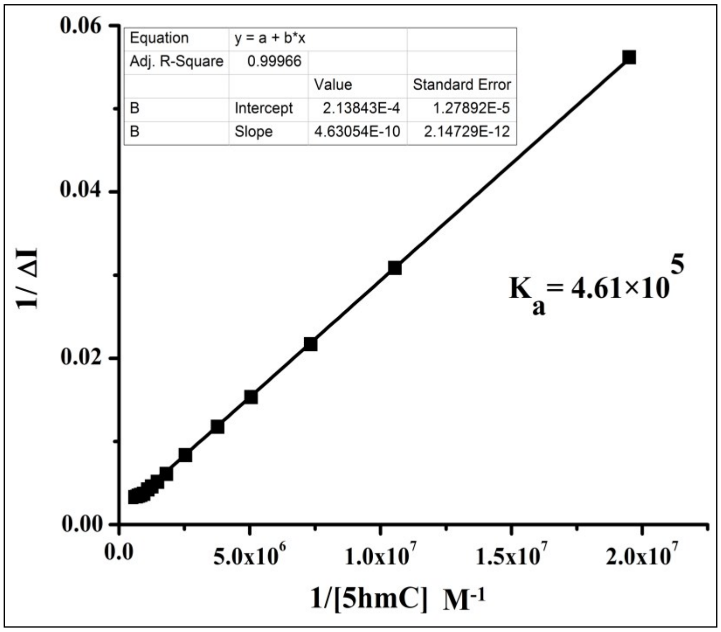

Fig. S7 Linear regression analysis for the calculation of association constant value by fluorescence titration method.

The association const. (K_a) of **CS-1** for sensing 5hmC was determined from the equation:

$$K_a = \text{intercept/slope.}$$

From the above mentioned linear fit graph (Figure S7) we get, intercept= 2.13843×10^{-4}, slope = 4.63054×10^{-10}. Thus we get, $K_a = (2.13843 \times 10^{-4}) / (4.63054 \times 10^{-10}) = 4.61 \times 10^5\ M^{-1}$.

Calculation of limit of detection (LOD) of CS-1 with 5hmC:

The detection limit of the chemosensor **CS-1** for 5hmC was calculated on the basis of fluorescence titration. To determine the standard deviation for the fluorescence intensity, the emission intensity of four individual receptors without 5hmC was measured by 10 times and the standard deviation of blank measurements was calculated.

The limit of detection (LOD) of **CS-1** for sensing 5hmC was determined from the following equation[5-6]:

$$LOD = K \times SD/S$$

Where K = 2 or 3 (we take 3 in this case); SD is the standard deviation of the blank receptor solution; S is the slope of the calibration curve.

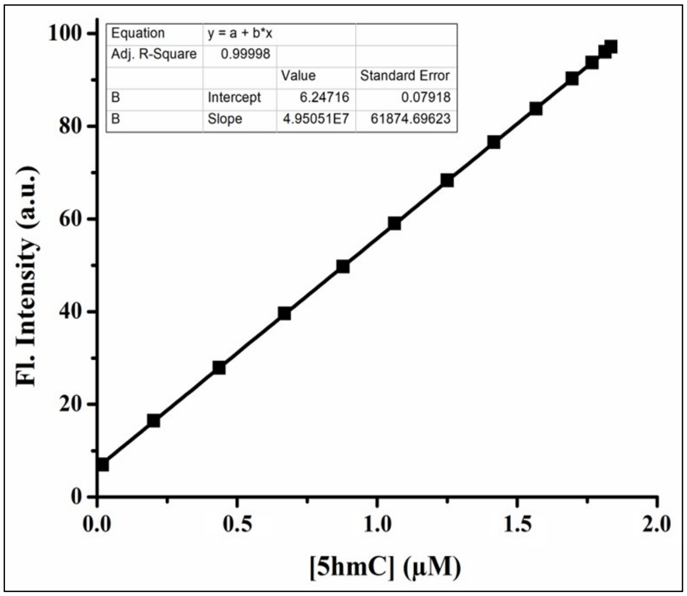

Fig. S8 Linear fit curve of **CS-1** at 565 nm with respect to 5hmC concentration.

For **CS-1** with 5hmC:

From the linear fit graph (Figure S8) we get slope = 4.95051×10^7, and SD value is 0.13587.

Thus using the above formula we get the Limit of Detection = 0.08×10^{-7} M, i.e. 8 nM. Therefore **CS-1** can detect 5hmC up to this very lower concentration by fluorescence study.

Selectivity studies:

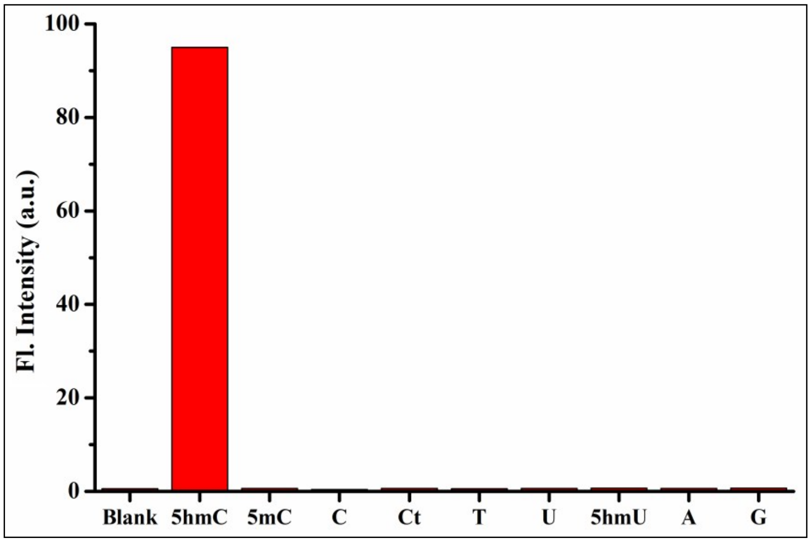

Fig. S9 Histogram representing fluorescence spectra of **CS-1** (1 μM) upon addition of 1.2 equiv. of guests at 565 nm (λ_{ex}= 325 nm) in H_2O–CH_3CN (15:1, v/v) [From left to right: **CS-1**, **CS-1** with– 5hmC, 5mC, cytosine, cytidine, thymine, uracil, 5hmU, adenine and guanine].

pH titration study:

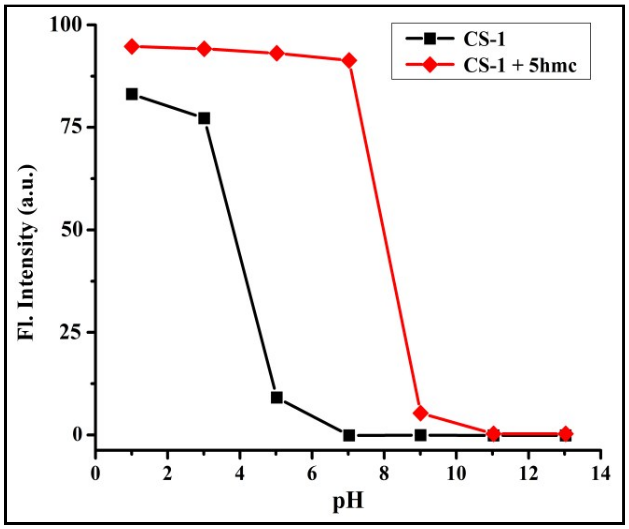

Fig. S10 Effect of pH on the fluorescence intensity of **CS-1** (1 μM) in the absence of 5hmC (black line) and in the presence of 5hmC (10 μM, red line).

^{1}H NMR titration spectrum of CS-1 with 5hmC:

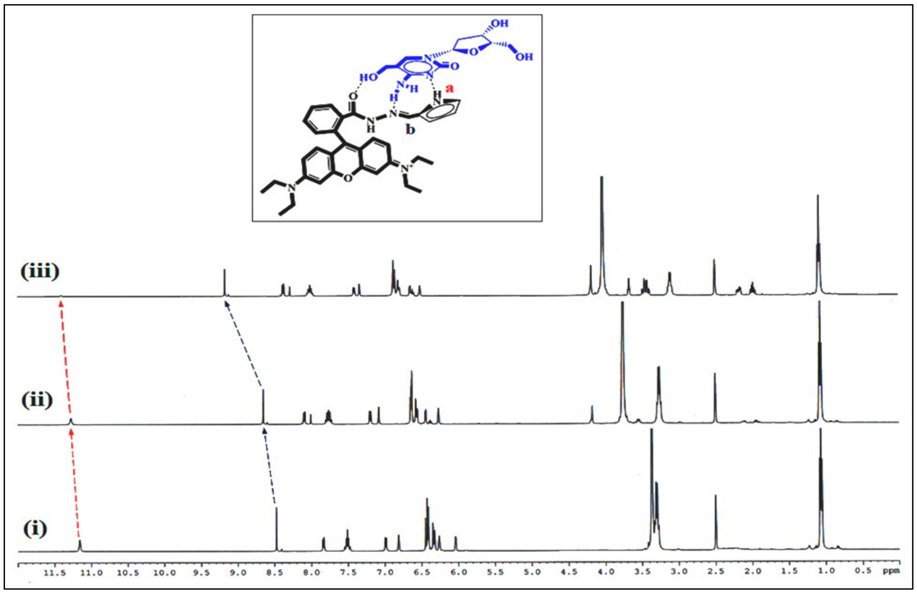

Fig. S11 ^{1}H NMR titration [400 MHz] spectra of **CS-1** in DMSO-d$_6$ at 25^{0}C and the corresponding changes after the gradual addition of 1 equiv. of 5hmC in D_2O; (i) only **CS-1**, (ii) **CS-1** + 0.5 equiv. of 5hmC, (iii) **CS-1** + 1 equiv. of 5hmC.

^{13}C NMR titration spectrum of CS-1 with 5hmC:

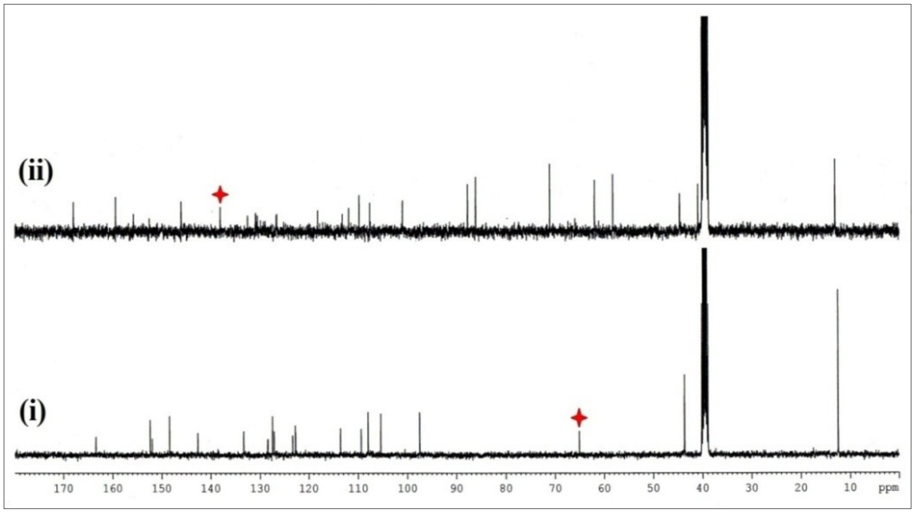

Fig. S12 ^{13}C NMR titration [400 MHz] spectra of **CS-1** in DMSO-d$_6$ at 25^{0}C and the corresponding changes after addition of 1 equiv. of 5hmC in D_2O; (i) only **CS-1**, (ii) **CS-1** + 1 equiv. of 5hmC. The red spot indicates the shifting of the spiro cyclic carbon peak from 65 ppm to 138 ppm in the open form.

Mass spectrum of CS-1–5hmC complex:

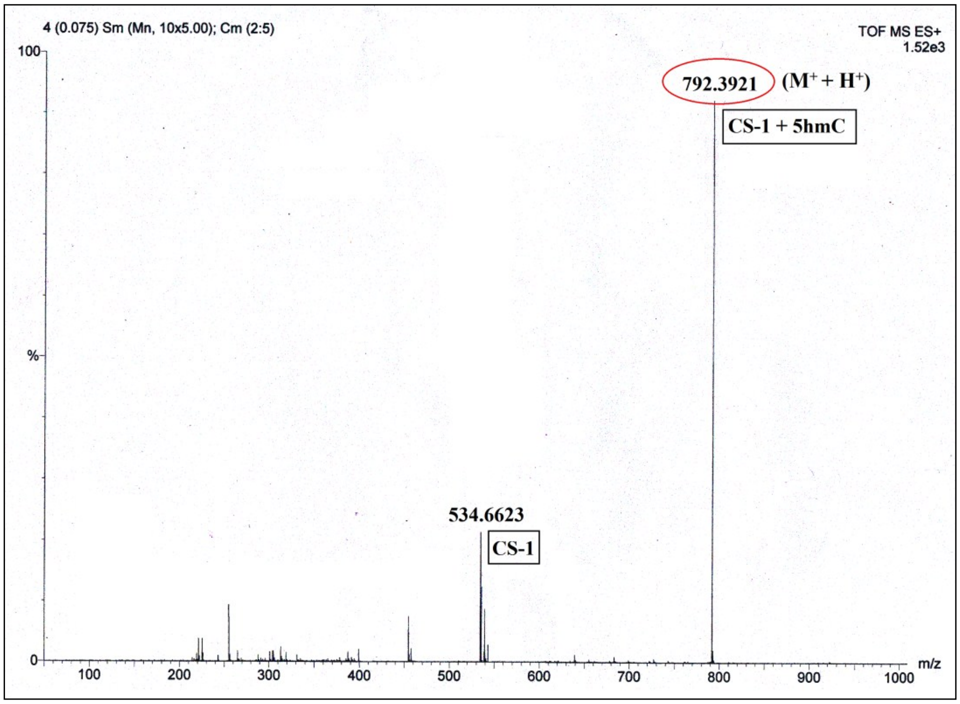

Fig. S13 HRMS of **CS-1**–5hmC complex.

DFT study:

Table S3. Details of the geometry optimization in Gaussian 09 program

**Details**	**CS-1**	**5hmC**	**CS-1–5hmC**
**Calculation method**	RB3LYP	RB3LYP	RB3LYP
**Basis set**	6-31G+**	6-31G+**	6-31G+**
**E(RB3LYP) (a.u.)**	-1702.905	-930.558	-2633.494
**Charge, Multiplicity**	0,1	0,1	1,1
**Solvent (CPCM)**	Water/Acetonitrile	Water	Water

Live cell imaging:

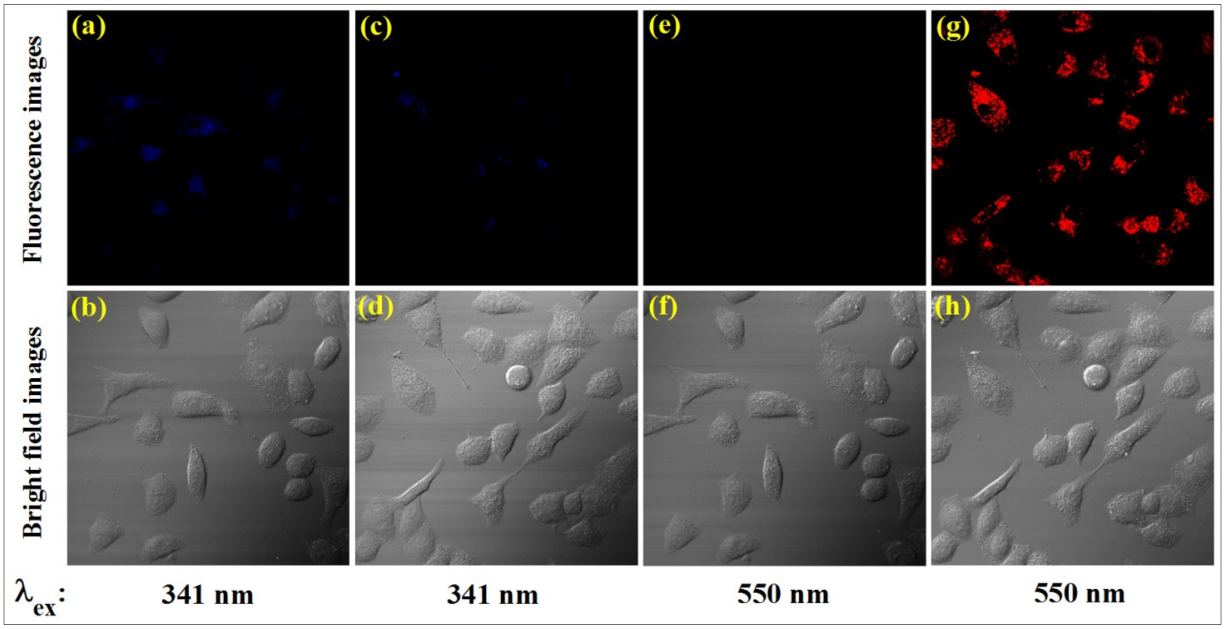

Fig. S14 Confocal microscopic images of A549 cells treated with **CS-1** and 5hmC. (a & e) Cells treated with only **CS-1** at 1 µM concentration. (b & f) Bright field images of (a) & (e), respectively. (c & g) Cells treated with **CS-1** and 5hmC at concentration 10 µM. (d & h) Bright field images of (c) & (g), respectively. All images were acquired with a 60X objective lens with the applied wavelengths: For (a), (b), (c), and (d), E_{ex}= 341 nm, E_{em}= 414 nm, filter used: DIDS; for (e), (f), (g), and (h) E_{ex}= 550 nm, E_{em}= 571 nm, filter used: Rhod-2.

Cytotoxicity Assay:

In vitro studies established the ability of the chemosensor **CS-1** to detect 5hmC in biological system with excellent selectivity. Human cancer cell A549 (ATCC No. CCL–185) were used as models. To materialize this objective, it is a prerequisite to assess the cytotoxic effect of **CS-1** and **CS-1**–5hmC complex on live cells. The well-established MTT assay[7] was adopted to study cytotoxicity of above mentioned complexes at varying concentrations. A cytotoxicity measurement for each experiment shows that the chemosensor **CS-1** does not have any toxicity on the tested cells and **CS-1**–5hmC complex does not exert any significant adverse effect on cell viability at tested concentrations (Figure S14).

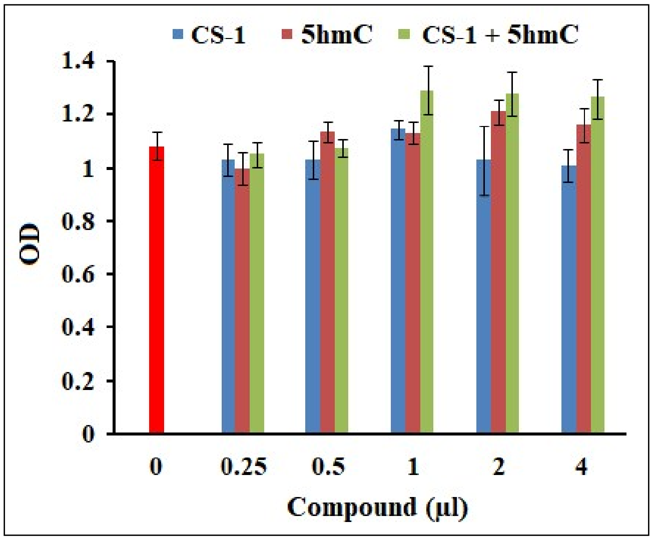

Fig. S15 MTT assay to determine the cytotoxic effect of **CS-1** and **CS-1**–5hmC complex on A549 cells (Human cancer cell A549, ATCC No CCL–185)

Live Cell Imaging:

Cell line and cell culture:

Cell culture: A549 cell (Human cell A549, ATCC No. CCL–185) lines were prepared from continuous culture in Dulbecco's Modified Eagle's Medium (DMEM, Sigma Chemical Co., St. Louis, MO) supplemented with 10% fetal bovine serum (Invitrogen), penicillin (100 µg/mL), and streptomycin (100 µg/mL). Cells were initially propagated in 75 cm^2 polystyrene, filter–capped tissue culture flask in an atmosphere of 5% CO_2 and 95% air at 37°C in CO_2 incubator. When the cells reached the logarithmic phase, the cell density was adjusted to 1.0×10^5 per/well in culture media. The cells were then used to inoculate in a glass bottom dish, with 1.0 mL (1.0×10^4 cells) of cell suspension in each dish. After cell adhesion, culture medium was removed. The cell layer was rinsed twice with phosphate buffered saline (PBS) (pH 7.0), and then treated according to the experimental need.

Cell imaging study:

For confocal imaging studies, 1 × 10^4 A549 cells in 1000 μL of medium, were seeded on sterile 35 mm glass bottom culture dish (ibidi GmbH, Germany), and incubated at 37°C in a CO_2 incubator for 10 hours. Then cells were washed with 500 μL DMEM followed by incubation with the chemosensor **CS-1** (1 μM) dissolved in 1000 μL DMEM at 37°C for 1 h in a CO_2 incubator and cells were washed thrice with phosphate buffered saline (PBS) (pH 7.0) to remove excess **CS-1** observed under an Olympus IX81 microscope equipped with a FV1000 confocal system using 1003 oil immersion Plan Apo (N.A. 1.45) objectives. Images obtained through section scanning were analyzed by DIDS with excitation at 341 nm monochromatic laser beams, and emission spectra were integrated over the range 414 nm (single channel). The cells were again incubated with 5hmC (10 μM) for 20 min and excess 5hmC was washed thrice with PBS (pH 7.0). Images were captured under microscope using Rhod-2 filter with excitation at 550 nm monochromatic laser beams, and emission spectra were integrated over the range 571 nm. For all images, the confocal microscope settings, such as transmission density, and scan speed, were held constant to compare the relative intensity of intracellular fluorescence.

Quantification of 5hmC in human cancer cells:

To quantify cellular level of 5hmC 10^7 A549 human cancer cells were harvested by centrifugation at 3000 rpm for 5 minutes followed by washing of the cell pellet with PBS buffer. Cells were again harvested following similar centrifugation. Cell pellets were suspended with 100 μL cold deionized water in order to lyse by the osmotic shock. Lysates were further centrifuged and the supernatant has been collected. The supernatant has been added with the chemosensor **CS-1** (1 μM) and the fluorescence signal was measured. The value of fluorescence intensity has been plotted to the standard curve in order to know the concentration of 5hmC in tested samples. All estimations have been done in triplicate (Table S4).

Table S4. Quantification of 5hmC in human cancer cell A549

Sample	**CS-1** used (μM)	Initial 5hmC used	Addition of exogenous 5hmC (μM)	Amount of 5hmC derived from fluorescence signal (μM)	Average fluorescence signal recovery (%)
1	1	5hmC present in 16.7 mm^3 A549 cell volume	0	0.033	–
	1		0	0.035	
	1		0	0.034	
2	1		1	1.028	99.4
	1		1	1.026	
	1		1	1.027	
3	1		3	4.018	99.6
	1		3	4.019	
	1		3	4.020	
4	1		5	5.012	99.5
	1		5	5.010	
	1		5	5.011	

Validation of the screening procedure:

The estimation of 5hmC was validated using different samples of A549 cancer cells. 10^4 of each cell suspension were centrifuged to collect the cells. The cells were resuspended with 10 mM PBS buffer (pH 7.0) followed by centrifugation. The cell pellets were lysed by osmotic shock with 100 μL ice cold deionised water. Supernatant were added with 1 μL **CS-1** and fluorescence signal were recorded. The fluorescence signal has been recorded for three independent samples of A549 cancer cells and all experiments were done in triplicate. The signal to noise ratio were obtained and the screening procedure were validated by calculating Z' score (Table S5).

Table S5. Optimization and validation of the screening procedure for 5hmC level in various samples of A549 human cancer cells using the chemosensor **CS-1**

Samples	Fluorescence Intensity			Mean	Standard Deviation	Signal*/Noise**	Z' score
	Set 1	Set 2	Set 3				
**Control**	0.19	0.21	0.20	0.20	0.01	----	----
**S1**	25	26	25	25.33	0.5773	126.66	0.92
**S2**	28	27	27	27.33	0.5773	136.66	0.93
**S3**	30	29	30	29.66	0.5773	148.33	0.94

*Fluorescence intensity for **CS-1**–5hmC interaction.

Fluorescence intensity for **CS-1.

References:

1. Yang, X. F.; Guo, X. Q.; Zhao, Y. B. *Anal. Chim. Acta* **2002**, *456*, 121–128.
2. Benesi, H. A.; Hildebrand, J. H. *J. Am. Chem. Soc.* **1949**, *71*, 2703.
3. Yang, C.; Liu, L.; Mu, T. -W.; Guo, Q. -X. *Anal. Sci*. **2000**, *16*, 537.
4. Shiraishi, Y.; Sumiya, S.; Kohno, Y.; Hirai, T. *J. Org. Chem.* **2008**, *73*, 8571.
5. Long, L.; Zhang, D.; Li, X.; Zhang, J.; Zhanga, C.; Zhou, L. *Anal. Chim. Acta* **2013**, *775*, 100.
6. Zhu, M.; Yuan, M.; Liu, X.; Xu, J.; Lv, J.; Huang, C.; Liu, H.; Li, Y.; Wang, S.; Zhu, D. *Org. Lett*. **2008**, *10*, 1481.
7. Ratha, J.; Majumdar, K. A.; Mandal, S. K.; Bera, R.; Sarkar, C.; Saha, B.; Mandal, C.; Saha, K. D.; Bhadra, R. *Mol. Cell. Biochem*. **2006**, *290*, 113.
